# Editorial: Advances in the Pathogenesis and Therapeutic Strategies for Nasopharyngeal Carcinoma

**DOI:** 10.3389/fonc.2021.647809

**Published:** 2021-03-09

**Authors:** Kai-Bin Yang, Cheng Xu, Yu-Pei Chen, Jan Baptist Vermorken, Brian O'Sullivan, Jun Ma

**Affiliations:** ^1^Zhongshan School of Medicine, Sun Yat-sen University, Guangzhou, China; ^2^State Key Laboratory of Oncology in South China, Department of Radiation Oncology, Collaborative Innovation Center for Cancer Medicine, Sun Yat-sen University Cancer Center, Guangzhou, China; ^3^Department of Medical Oncology, Antwerp University Hospital, Edegem, Belgium; ^4^Faculty of Medicine and Health Sciences, University of Antwerp, Antwerp, Belgium; ^5^Department of Radiation Oncology, Princess Margaret Cancer Center, University of Toronto, Toronto, ON, Canada

**Keywords:** nasopharyngeal carcinoma, epstein barr virus, pathogenesis, therapeutic strategies, concurrent chemoradiotherapy, recurrence

## Background

Originating from the nasopharyngeal epithelium, nasopharyngeal carcinoma (NPC) is an Epstein-Barr virus (EBV)-related cancer that features an extremely uneven geographical and racial distribution. Its incidence varies widely from 30 in 100,000 individuals in endemic areas to <1 in 100,000 individuals within mainly white populations in non-endemic areas (1–3).

Given the anatomic constraints and the high radiosensitivity of NPC, radiotherapy (RT) is currently the mainstay of definitive treatment for non-metastatic disease (4). Over the past three decades, the management of NPC patients and, accordingly, their prognosis has shown great improvement (5). Firstly, the innovations in RT technology and the extensive application of intensity-modulated radiotherapy (IMRT) with advantageous dose distribution has improved locoregional control and reduced toxic effects on adjacent organs (6–8). In parallel, the prognosis was further improved with the addition of platinum-based chemotherapy to RT, especially for locoregionally advanced NPC (LANPC), probably owing to the improved distant control and enhanced sensitivity to RT. The survival benefits and safety of concurrent chemoradiotherapy (CCRT) and neoadjuvant chemotherapy (NACT) for LANPC have been confirmed by several clinical trials (9–15), and NACT+CCRT is currently the recommended therapy for LANPC in international guidelines (16, 17). However, certain controversies still exist in the management and prognosis of NPC, and a considerable number of studies have focused on tackling them. The present article sheds light on these challenges and the solutions proposed by various research groups.

## Limitations in RT

Although the improved locoregional control and reduced toxicities has been achieved in the era of IMRT, successful RT still relies on precise delineation and exact dose delivery to the target volume, which is time-consuming and susceptible to inter-observer variability despite the establishment of international contouring guidelines (18). In a retrospective study by Iacovelli et al., the predictive effect of the dose and volume parameters of RT for non-metastatic NPC was evaluated. Since there is a scarcity of medical evidence in non-endemic areas, this study can provide physicians and investigators with valuable information on the radiotherapy of patients with NPC. Automated delineation through deep-learning algorithms is an appealing option to overcome the shortcomings of artificial contouring. A fully-automated delineation method based on dual-sequence MRI of NPC was proposed by Ye et al. Integrating the different image features of NPC in T1W and T2W images using a dense connectivity embedding U-net, their method demonstrated efficient, accurate, and robust performance within an external validation dataset. In particular, its fully automated design makes it convenient to use.

## The Challenge of Recurrence

Another challenge in NPC treatment is recurrence, especially in patients with advanced disease, even after intensive treatment. After definitive IMRT, 5–10% of NPC patients develop locoregional recurrence, and most of them develop in the first 5 years of follow-up, especially in the first 2 years (19). Using 2 years as a cut-off, the recurrences were classified into early type and late type, and the clinical characteristics and prognostic factors of early vs. late relapses were investigated in a retrospective study by Li F. et al. Surveillance following anti-cancer treatment is another important strategy for tackling the high failure rate of locoregional control. The NPC surveillance guidelines provided by the National Comprehensive Cancer Network and European Society for Medical Oncology were evaluated by Zhou et al. in a retrospective study, and their results showed that most recurrences would be missed if either of the two guidelines was strictly followed, indicating an urgent need for improved surveillance algorithms. Additionally, the suspicion of a clinically recurrent event can be confused with complications associated with radiotherapy. For example, cervical spine osteoradionecrosis may be mistaken as metastasis due to the increased radiotracer uptake on a bone scan. In their retrospective study, Zhong et al. demonstrated the additional value of MRI in differentiating between cervical spine osteoradionecrosis and metastasis detected by bone scan, further enabling the early detection and treatment of recurrent diseases and the elimination of unnecessary intensive therapy for benign lesions. Besides surveillance, treatment of recurrences also remains a problem. Endoscopic nasopharyngectomy is one of the treatment options for local recurrence after radiotherapy. Site-specific and sinonasal-related quality of life (QoL) was shown to be impaired immediately after salvage nasopharyngectomy and to gradually recover to preoperative levels during long-term follow-up in a prospective study by Li W. et al.. Their results confirmed that endoscopic nasopharyngectomy is a valuable management option for local recurrence and indicated that gross-total resection was superior to subtotal resection considering the postoperative QoL.

## Impaired QoL and Toxicity

Another current challenge in NPC is the substantial burden of long-term toxicity and impaired QoL in survivors after successful anti-cancer treatment. McDowell et al. have provided a detailed review of the toxicity and long-term QoL data in prospective studies of chemotherapy and RT from both endemic and non-endemic areas. Factors affecting long-term QoL, the unmet needs of NPC survivors in the contemporary era, and potential and promising strategies to reduce the toxicity burden were all highlighted. Their review provided a profile of unmet needs in NPC survivors and, additionally, pointed out two major shortcomings in the presently available data to provide valuable guidance for future research. One of the shortcomings was that the vast majority of QoL and toxicity data was reported from the clinician's perspective, which may result in unintentional underestimation of symptoms and their severity. The second shortcoming was that statistically determined, rather than clinically meaningful, differences were more commonly reported.

Since more intensive therapies generally facilitate disease control at the expense of more severe toxicity, striking a balance between disease control and toxicity is key to the optimal treatment of all cancers, including NPC. To assist in the comprehensive evaluation of both the efficacy and toxicities of different cancer treatment options, the ASCO-VF and ESMO-MCBS frameworks were proposed (20, 21). In a field test of these two frameworks in the context of NPC, Zhang et al. reported significant variations in the toxicity data reported by different trials and inconsistent scores generated by ASCO-VF for treatments that were defined as “substantial clinical benefit” in ESMO-MCBS. Thus, there seems to be some inconsistency between the two frameworks, which requires more attention in the future. Given the additional toxicity and economic burden of CCRT and NACT, identification of the potential beneficiaries for these treatments to help avoid unnecessary chemotherapy is another feasible measure to achieve better balance between treatment efficacy and toxicity. In their Phase 2 Multicenter Clinical Trial, Huang et al. evaluated the efficacy of CCRT in stage II NPC in the IMRT era. In contrast to the results in another phase 3 trial adopting conventional 2-dimensional radiotherapy published previously (22), their results showed that CCRT failed to further improve the prognosis of stage II NPC compared with IMRT alone. Thus, additional concurrent chemotherapy may be unnecessary for stage II NPC in the IMRT era. Prediction of the potential beneficiaries for NACT in LANPC has also been extensively studied via two relevant retrospective studies from endemic areas. One of the studies targeted the entire LANPC population and established a prognostic index model that uses gender, T status, N status, LDH level, and EBV-DNA level to identify high-risk patients for additional NACT (Sun et al.), while the other one focused on the usually excluded T3N0-1 and T4N0 subgroups. Combining real-world and clinical trial data together, a risk stratification model including gender and EBV-DNA level was generated and validated using recursive partitioning analysis (Xu et al.). Notably, gender and EBV-DNA level were identified as risk factors in both studies, indicating their close association with the patient's prognosis.

## Pathogenesis of NPC

Besides the clinical studies on therapeutic strategies, research effort has also been devoted to deepening our understanding of the pathogenesis of NPC, with the intention of promoting the development of novel screening strategies and targeted therapies with high efficacy and low toxicity, especially to better treat NPC recurrences and distant metastasis. Since NPC is consistently related to the EBV infection in endemic areas, extensive research has focused on the role of multiple viral latent gene products, such as EBNA1, LMP1, and LMP2, in the malignant transformation of the nasopharyngeal epithelium and EBV-targeting therapy for NPC. These studies and their findings have been reviewed in detail by Hau et al., who report that EBV-targeting therapeutic strategies for NPC include targeting EBV latent proteins and switching the latent cycle of EBV to the lytic cycle. Although no specific EBV-targeting therapeutic strategy has been approved for NPC at present, this strategy remains promising and appealing for this EBV-related malignancy.

Abnormal function of proto-oncogenes and tumor suppressor genes form a common pathogenic mechanism in all cancers, including NPC. Accordingly, Qin et al. reported a heterozygous mutation of p53, a well-known tumor suppressor gene, and its oncogenic effect through activation of the PI3K-Akt signaling pathway in NPC cells. Additionally, Wang et al. found that the internal ribosome entry sites of Bmi1, a proto-oncogene in polycomb-repressive complex 1, mediated its cap-independent translation in NPC cells.

Extensive application of high-throughput sequencing techniques has greatly promoted research on the pathogenesis of cancer. With the help of these techniques, the altered intestinal flora in NPC patients and circRNA expression profiles in NPC were revealed by Jiang et al. and Yang et al., respectively. While the alteration of flora might be useful in early screening and individualized prevention and treatment of NPC, the differentially expressed circRNAs and their target pathways might provide novel targets for NPC therapy.

## Other Limitations

A notable shortcoming in research on NPC is that it is poorly studied in children and adolescents, probably due to its rarity in younger age groups. In a large cohort study of childhood and adolescent NPC treated with IMRT, the clinical significance of plasma EBV-DNA was confirmed (Qiu et al.). However, more research effort is required to facilitate optimization of treatment for patients from this group, as the findings for adulthood NPC may not be translatable to children and adolescents with NPC.

## Conclusion

Despite the advances in research on the management and treatment of NPC over the past three decades, toxicity, recurrence, and standardization of RT remain major challenges, along with a lack of research about NPC in children and adolescents. Nonetheless, advances are being witnessed in research on the pathogenesis and therapeutic strategies of NPC. With concerted global research efforts, the current obstacles in NPC treatment will be overcome, and we are bound to win the battle against NPC eventually.

## Author Contributions

K-BY and CX drafted the manuscript. JM, JV, and BO'S revised the manuscript. All authors approved the submission.

## Conflict of Interest

The authors declare that the research was conducted in the absence of any commercial or financial relationships that could be construed as a potential conflict of interest.

## References

[1] Wei KR Zheng RS Zhang SW Liang ZH Li ZM Chen WQ. Nasopharyngeal carcinoma incidence and mortality in China, 2013. Chin J Cancer. (2017) 36:90. 10.1186/s40880-017-0257-9 29122009 PMC5679327

[2] Bray F Ferlay J Soerjomataram I Siegel RL Torre LA Jemal A. Global cancer statistics 2018: GLOBOCAN estimates of incidence and mortality worldwide for 36 cancers in 185 countries. CA Cancer J Clin. (2018) 68:394–424. 10.3322/caac.21492 30207593

[3] Ferlay J Colombet M Soerjomataram I Mathers C Parkin DM Piñeros M. Estimating the global cancer incidence and mortality in 2018: GLOBOCAN sources and methods. Int J Cancer. (2019) 144:1941–53. 10.1002/ijc.31937 30350310

[4] Peng H Chen L Chen YP Li WF Tang LL Lin AH. The current status of clinical trials focusing on nasopharyngeal carcinoma: a comprehensive analysis of ClinicalTrials.gov database. PLoS ONE. (2018) 13:e0196730. 10.1371/journal.pone.0196730 29718970 PMC5931495

[5] Chen YP Chan ATC Le QT Blanchard P Sun Y Ma J. Nasopharyngeal carcinoma. Lancet. (2019) 394:64–80. 10.1016/S0140-6736(19)30956-0 31178151

[6] Peng G Wang T Yang KY Zhang S Zhang T Li Q. A prospective, randomized study comparing outcomes and toxicities of intensity-modulated radiotherapy vs. conventional two-dimensional radiotherapy for the treatment of nasopharyngeal carcinoma. Radiotherap Oncol. (2012) 104:286–93. 10.1016/j.radonc.2012.08.013 22995588

[7] Zhang B Mo Z Du W Wang Y Liu L Wei Y. Intensity-modulated radiation therapy versus 2D-RT or 3D-CRT for the treatment of nasopharyngeal carcinoma: a systematic review and meta-analysis. Oral Oncol. (2015) 51:1041–6. 10.1016/j.oraloncology.2015.08.005 26296274

[8] Co J Mejia MB Dizon JM. Evidence on effectiveness of intensity-modulated radiotherapy versus 2-dimensional radiotherapy in the treatment of nasopharyngeal carcinoma: meta-analysis and a systematic review of the literature. Head Neck. (2016) 38(Suppl. 1):E2130–42. 10.1002/hed.23977 25546181

[9] Al-Sarraf M LeBlanc M Giri PG Fu KK Cooper J Vuong T. Chemoradiotherapy versus radiotherapy in patients with advanced nasopharyngeal cancer: phase III randomized Intergroup study 0099. J Clin Oncol. (1998) 16:1310–7. 10.1200/JCO.1998.16.4.1310 9552031

[10] Lin JC Jan JS Hsu CY Liang WM Jiang RS Wang WY. Phase III study of concurrent chemoradiotherapy versus radiotherapy alone for advanced nasopharyngeal carcinoma: positive effect on overall and progression-free survival. J Clin Oncol. (2003) 21:631–7. 10.1200/JCO.2003.06.158 12586799

[11] Sun Y Li WF Chen NY Zhang N Hu GQ Xie FY. Induction chemotherapy plus concurrent chemoradiotherapy versus concurrent chemoradiotherapy alone in locoregionally advanced nasopharyngeal carcinoma: a phase 3, multicentre, randomised controlled trial. Lancet Oncol. (2016) 17:1509–20. 10.1016/S1470-2045(16)30410-7 27686945

[12] Li WF Chen NY Zhang N Hu GQ Xie FY Sun Y. Concurrent chemoradiotherapy with/without induction chemotherapy in locoregionally advanced nasopharyngeal carcinoma: long-term results of phase 3 randomized controlled trial. Int J Cancer. (2019) 145:295–305. 10.1002/ijc.32099 30613964

[13] Cao SM Yang Q Guo L Mai HQ Mo HY Cao KJ. Neoadjuvant chemotherapy followed by concurrent chemoradiotherapy versus concurrent chemoradiotherapy alone in locoregionally advanced nasopharyngeal carcinoma: a phase III multicentre randomised controlled trial. Europ J Cancer. (2017) 75:14–23. 10.1016/j.ejca.2016.12.039 28214653

[14] Frikha M Auperin A Tao Y Elloumi F Toumi N Blanchard P. A randomized trial of induction docetaxel-cisplatin-5FU followed by concomitant cisplatin-RT versus concomitant cisplatin-RT in nasopharyngeal carcinoma (GORTEC 2006-02). Ann Oncol. (2018) 29:731–6. 10.1093/annonc/mdx770 29236943

[15] Zhang Y Chen L Hu GQ Zhang N Zhu XD Yang KY. Gemcitabine and cisplatin induction chemotherapy in nasopharyngeal carcinoma. N Engl J Med. (2019) 381:1124–35. 10.1056/NEJMoa1905287 31150573

[16] Colevas AD Yom SS Pfister DG Spencer S Adelstein D Adkins D. NCCN guidelines insights: head and neck cancers, Version 1.2018. J Natl Compr Canc Netw. (2018) 16:479–90. 10.6004/jnccn.2018.0026 29752322

[17] National Comprehensive Cancer Network. NCCN Guidelines for Head and Neck Cancers (Version 1.2021) Available online at: https://www.nccn.org/professionals/physician_gls/pdf/head-and-neck.pdf (accessed January 20, 2021).

[18] Lee AW Ng WT Pan JJ Poh SS Ahn YC AlHussain H. International guideline for the delineation of the clinical target volumes (CTV) for nasopharyngeal carcinoma. Radiotherap Oncol. (2018) 126:25–36. 10.1016/j.radonc.2017.10.032 29153464

[19] Lee AW Ma BB Ng WT Chan AT. Management of nasopharyngeal carcinoma: current practice and future perspective. J Clin Oncol. (2015) 33:3356–64. 10.1200/JCO.2015.60.9347 26351355

[20] Cherny NI Dafni U Bogaerts J Latino NJ Pentheroudakis G Douillard JY. ESMO-magnitude of clinical benefit scale version 1.1. Ann Oncol. (2017) 28:2340–66. 10.1093/annonc/mdx310 28945867

[21] Schnipper LE Davidson NE Wollins DS Blayney DW Dicker AP Ganz PA. Updating the american society of clinical oncology value framework: revisions and reflections in response to comments received. J Clin Oncol. (2016) 34:2925–34. 10.1200/JCO.2016.68.2518 27247218

[22] Chen QY Wen YF Guo L Liu H Huang PY Mo HY. Concurrent chemoradiotherapy vs radiotherapy alone in stage II nasopharyngeal carcinoma: phase III randomized trial. J Natl Cancer Inst. (2011) 103:1761–70. 10.1093/jnci/djr432 22056739

